# Impact of frailty on clinical outcomes and resource use following emergency general surgery in the United States

**DOI:** 10.1371/journal.pone.0255122

**Published:** 2021-07-23

**Authors:** Joseph Hadaya, Yas Sanaiha, Catherine Juillard, Peyman Benharash

**Affiliations:** 1 Division of Cardiac Surgery, Department of Surgery, David Geffen School of Medicine at UCLA, Los Angeles, California, United States of America; 2 Division of General Surgery, Department of Surgery, David Geffen School of Medicine at UCLA, Los Angeles, California, United States of America; Emory University School of Medicine, UNITED STATES

## Abstract

**Background:**

Frailty has been recognized as an independent risk factor for inferior outcomes, but its effect on emergency general surgery (EGS) is understudied.

**Objective:**

The purpose of the present study was to define the impact of frailty on risk-adjusted mortality, non-home discharge, and readmission following EGS operations.

**Methods:**

Adults undergoing appendectomy, cholecystectomy, small bowel resection, large bowel resection, repair of perforated ulcer, or laparotomy within two days of an urgent admission were identified in the 2016–2017 Nationwide Readmissions Database. Frailty was defined using diagnosis codes corresponding to the Johns Hopkins Adjusted Clinical Groups frailty indicator. Multivariable regression was used to study in-hospital mortality and non-home discharge by operation, and Kaplan Meier analysis to study freedom from unplanned readmission at up to 90-days follow-up.

**Results:**

Among 655,817 patients, 11.9% were considered frail. Frail patients most commonly underwent large bowel resection (37.3%) and cholecystectomy (29.2%). After adjustment, frail patients had higher mortality rates for all operations compared to nonfrail, including those most commonly performed (11.9% [95% CI 11.4–12.5%] vs 6.0% [95% CI 5.8–6.3%] for large bowel resection; 2.3% [95% CI 2.0–2.6%] vs 0.2% [95% CI 0.2–0.2%] for cholecystectomy). Adjusted non-home discharge rates were higher for frail compared to nonfrail patients following all operations, including large bowel resection (68.1% [95% CI 67.1–69.0%] vs 25.9% [95% CI 25.2–26.5%]) and cholecystectomy (33.7% [95% CI 32.7–34.7%] vs 2.9% [95% CI 2.8–3.0%]). Adjusted hospitalization costs were nearly twice as high for frail patients. On Kaplan-Meier analysis, frail patients had greater unplanned readmissions (log rank P<0.001), with 1 in 4 rehospitalized within 90 days.

**Conclusions:**

Frail patients have inferior clinical outcomes and greater resource use following EGS, with the greatest absolute differences following complex operations. Simple frailty assessments may inform expectations, identify patients at risk of poor outcomes, and guide the need for more intensive postoperative care.

## Introduction

Operative emergencies in acute care surgery are associated with substantial risk of mortality and rehospitalization [1–3]. Emergency general surgery (EGS) operations are often performed in patients with severely deranged physiology, and occasionally in those with hemodynamic compromise and end organ dysfunction [4,5]. The underlying pathology in this cohort is often acute in presentation, limiting the body’s compensatory responses. Several factors including age, operative type, and burden of comorbidities are thought to impact the outcomes of EGS operations [2,4,6,7].

Traditional risk factors such as advanced age have been recognized to inadequately predict outcomes following complex operations [8–10]. Recently, several investigators have reported frailty to influence postoperative outcomes including death [10–14]. Often associated with accumulation of chronic conditions, frailty is generally considered as the inability to withstand physiologic stressors [15–17]. In the setting of EGS operations, Murphy et al. used the National Surgical Quality Improvement Program (NSQIP) database to identify frailty using the modified frailty index (mFI) and found frailty to adversely impact EGS outcomes in those >40 years of age [18]. However, applicability of these findings is limited by low participation rates in NSQIP (12% of hospitals performing surgery in 2013) and only 3.6% of the study cohort classified as highly frail by the mFI [19]. Furthermore, hospitals participating in NSQIP have greater case volumes and hospital beds, are more frequently academically-affiliated and less commonly critical access hospitals [19]. Thus, data sources with more uniform participation may provide a more accurate landscape of outcomes following EGS, particularly in high risk cohorts such as the frail.

While a universal definition for frailty is lacking, several instruments ranging from intricate psychomotor testing to administrative algorithms have been employed to diagnose and quantify this state [20–24]. Many frailty tests are resource intensive and cannot be administered in the non-elective setting [23,24]. Coding-based scoring systems have garnered attention as methods to identify frailty using administrative data. The Johns Hopkins Adjusted Clinical Group cluster of diagnoses has recently been implemented as a coding-based method to identify frail patients in surgical patients, including those undergoing head and neck operations and cardiac surgery [25–28]. This binary system has several advantages, including ease of implementation (as it is solely derived from administrative data), lack of additional resources required for data collection, and inclusion of characteristics that do not typically overlap with postoperative complications.

We examined the impact of frailty as assessed by the Johns Hopkins frailty index on clinical outcomes and resource use following EGS operations in all adults using the Nationwide Readmissions Database (NRD), a widely inclusive administrative database. We hypothesized frailty to be independently associated with increased mortality, length of stay, hospitalization costs, rates of non-home discharge as well as readmissions across common EGS operations.

## Methods

### Data source and cohort definitions

The present study was a retrospective cohort study using the 2016–2017 NRD. The NRD is the largest, all-payer, national readmissions database and is maintained by the Agency for Healthcare Research and Quality (AHRQ) as part of the Healthcare Cost and Utilization Project (HCUP) [29]. The NRD samples 28 State Inpatient Databases annually and represents approximately 58% of all hospitalizations in the United States [29]. Patient-specific linkage numbers allow patients to be tracked across inpatient hospitalizations within each calendar year.

*International Classification of Disease*, *Tenth Edition*, *Procedure Coding System* (ICD-10-PCS) codes were used to identify patients undergoing one of the following EGS operations: large bowel resection, small bowel resection, repair of perforated ulcer, cholecystectomy, appendectomy, and lysis of adhesions. These procedures were chosen due to their frequency and clinical relevance to general surgery practice [30]. If multiple EGS procedures were tabulated for a patient, the primary operation was considered the procedure with the greatest probability of mortality [30].

Patients under the age of 18 and those admitted on an elective basis were excluded from further study. Patients with an admission for injury or trauma were excluded using *International Classification of Disease*, *Tenth Revision*, *Clinical Modification* (ICD-10-CM) codes validated by the National Center for Health Statistics [31]. Patients with missing data for age, sex, admission type, and in-hospital mortality were excluded. To maintain a consistent definition of EGS operations, only operations performed on hospital days 0, 1, or 2 were considered [30].

Patients were divided into frail (*Frail*) and nonfrail (*Nonfrail*) cohorts, with frailty identified by the presence of at least one frailty-defining diagnosis as reported by Neiman et al [25]. ICD-10-CM codes were used to identify relevant diagnoses, which were derived from the validated Johns Hopkins Adjusted Clinical Groups (ACG) frailty-defining diagnoses indicator. This binary indicator categorizes frailty-defining diagnoses into malnutrition, dementia, impaired vision, decubitus ulcer, incontinence, weight loss, falls, difficulty walking, poverty, and barriers to healthcare access (S1 Table) [25]. Derivatives of the ACG have been extensively used in medical and surgical studies of frailty in administrative databases [25–28].

### Variable definitions and study outcomes

Patient and hospital characteristics included age, sex, admission type, primary payer, income quartile, and hospital teaching status. Each hospital’s annual emergency general surgery volume was calculated, and hospitals were divided into volume low-, medium-, and high-volume tertiles based on the annual aggregate EGS caseload for each center. The Elixhauser Comorbidity Index, a validated composite score of 30 chronic comorbidities, was used to quantify patient comorbidities [32].

Mortality was defined as death during the index hospitalization. Non-home discharge was defined as discharge to an acute hospital, intermediate care facility, or skilled nursing facility. Readmission was defined as unplanned rehospitalization among patients surviving to index discharge. Hospital costs were calculated from charges using hospital-specific cost-to-charge ratios reported by the AHRQ and adjusted for inflation to 2017 using the Bureau of Labor Statistics Consumer Price Index [29,33].

The primary outcomes of the study were mortality at the index hospitalization, non-home discharge, and 30-day unplanned readmission rates. Several secondary outcomes included postoperative length of stay and hospitalization costs.

### Statistical analysis

Categorical variables were reported as frequency and percent and continuous variables as mean and standard deviation, or median and interquartile range if non-normally distributed. Chi-squared and adjusted Wald t-tests were used to compare patient and hospital characteristics.

Multivariable logistic and linear models were used to identify independent associations between outcomes and frailty and covariates. A generalized linear regression model with gamma error distribution and log-link function was used to study costs. Hospital-specific discharge weights were used to obtain survey-weighted estimates that account for clustering [29]. Covariates remaining after backward stepwise elimination and those deemed clinically relevant were included in final models. Interaction terms between frailty and EGS operation type, and EGS operation type and hospital teaching status were included in all models. Models were evaluated using the receiver operating characteristics curve and Akaike information criterion. Following each regression, estimates were calculated using the Stata *margins* command. Adjusted outcomes are reported as estimates with 95% confidence interval (95% CI). Kaplan-Meier survival analysis with log rank test was used to compare occurrence of unplanned readmissions by cohort. Follow-up time was constrained by the structure of NRD, as patients are followed through the end of each calendar year.

This study was deemed exempt by the Institutional Review Board at the University of California, Los Angeles. Statistical analysis was performed with Stata 16.0 (StataCorp, College Station, TX). Statistical significance was set at α<0.05.

## Results

### *Frail* and *Nonfrail* cohort characteristics

Of an estimated 655,817 patients, 78,093 (11.9%) were considered *Frail*. Patients in the *Frail* cohort were older, with 65.0% of *Frail* >65 years of age, compared to 32.4% of *Nonfrail* (Table 1). The *Frail* cohort had a greater composite burden of comorbidities, with an Elixhauser Comorbidity Score >2 in 76.7% vs 34.9% in the *Nonfrail* cohort. There were greater rates of all examined comorbidities in the *Frail* cohort, including congestive heart failure, chronic lung disease, chronic liver disease, and end stage renal disease. The predominant primary payer in the *Frail* cohort was Medicare (68.3%), compared to private insurance (37.9%) in *Nonfrail*. Most patients were treated at teaching hospitals (64.1% of *Frail* vs 61.3% of *Nonfrail*). The most common operations performed were cholecystectomy (63.9% of *Nonfrail* and 29.2% of *Frail*) and large bowel resection (14.7% of *Nonfrail* and 37.3% of *Frail*, Table 1). A majority of patients underwent surgery on the day of admission (Table 1).

**Table 1:** Demographics and characteristics of patients undergoing EGS operations.

	*Nonfrail* (N = 577,724)	*Frail* (N = 78,093)	P-value
Age^a^	55 (39–68)	71 (59–81)	<0.001
Age ≥ 65	187,180 (32.4)	50,731 (65.0)	<0.001
Female	348,866 (60.4)	42,468 (54.4)	<0.001
Primary Payer			<0.001
Private	218,500 (37.9)	13,315 (17.1)	
Medicare	202,354 (35.1)	53,313 (68.3)	
Medicaid	101,023 (17.5)	7,777 (10.0)	
Other Payer^b^	55,069 (9.5)	3,628 (4.7)	
Income Quartile			0.24
Fourth (Highest)	108,106 (18.5)	14,237 (18.9)	
Third	141,748 (24.9)	19,002 (24.7)	
Second	157,274 (27.6)	21,294 (27.7)	
First (Lowest)	162,727 (28.6)	22,410 (29.1)	
Day of Operation			<0.001
Hospital Day 0	238,064 (41.2)	33,117 (42.4)	
Hospital Day 1	213,648 (37.0)	25,649 (32.8)	
Hospital Day 2	126,012 (21.8)	19,326 (24.8)	
Primary Operation			<0.001
Large Bowel Resection	85,102 (14.7)	29,099 (37.3)	
Small Bowel Resection	46,641 (8.1)	13,983 (17.9)	
Cholecystectomy	369,304 (63.9)	22,837 (29.2)	
Repair of Perforated Ulcer	10,151 (1.8)	4,491 (5.8)	
Lysis of Adhesions	35,729 (6.2)	5,207 (6.7)	
Appendectomy	30,796 (5.3)	2,476 (3.2)	
Hospital Teaching Status	354,275 (61.3)	50,065 (64.1)	<0.001
Hospital Volume			0.070
Low	183,077 (31.7)	24,229 (31.0)	
Medium	184,578 (32.0)	24,371 (31.2)	
High	210,069 (36.4)	29,492 (37.8)	
Elixhauser Comorbidity Index			
≤ 2	375,885 (65.1)	18,207 (23.3)	<0.001
> 2	201,839 (34.9)	59,886 (76.7)	<0.001
Comorbidities			
Chronic Liver Disease	42,415 (7.3)	7,407 (9.5)	<0.001
Chronic Lung Disease	82,153 (14.2)	17,698 (22.7)	<0.001
Coagulopathy	22,494 (3.9)	10,270 (13.2)	<0.001
Congestive Heart Failure	31,030 (5.4)	13,380 (17.1)	<0.001
Coronary Artery Disease	58,941 (10.2)	15,755 (20.2)	<0.001
Diabetes	102,620 (17.8)	19,110 (24.5)	<0.001
End Stage Renal Disease	9,178 (1.6)	3,684 (4.7)	<0.001
Hypertension	263,448 (45.6)	48,942 (62.7)	<0.001
Hypothyroidism	58,329 (10.1)	11,860 (15.2)	<0.001
Metastatic Cancer	15,438 (2.7)	6,361 (8.2)	<0.001
Non-metastatic Cancer	22,516 (3.9)	7,458 (9.6)	<0.001
Peripheral Vascular Disease	29,610 (5.1)	11,072 (14.2)	<0.001
Pulmonary Circulatory Disorder	8,458 (1.5)	3,739 (4.8)	<0.001
Rheumatologic Disorder	12,713 (2.2)	2,851 (3.7)	<0.001

^a^Age reported as median and interquartile range. Remainder of characteristics reported as frequency and percentage.

^b^Other payer includes self-pay, and uninsured.

### Clinical outcomes in *Frail* vs *Nonfrail* patients by operation

**Fig 1:**
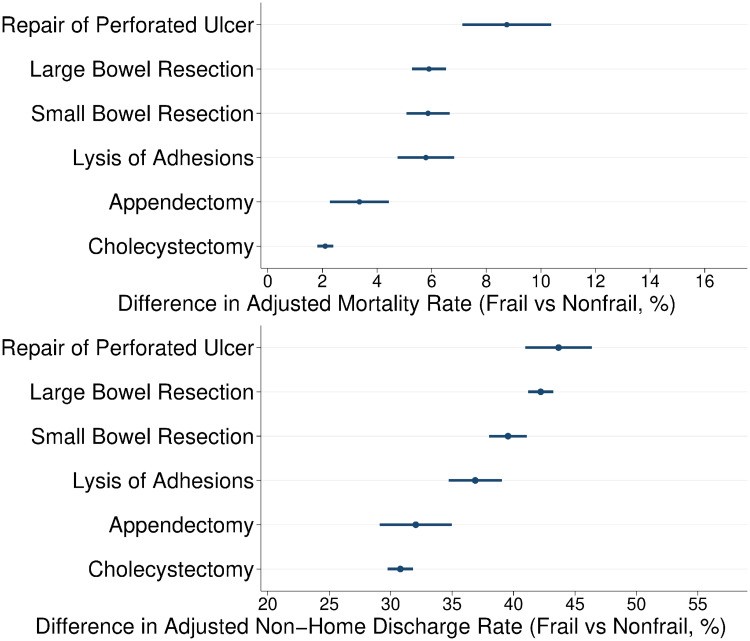
Differences in adjusted rates of mortality (top) and non-home discharge (bottom) in *Frail* versus *Nonfrail* patients. Estimate represents mean absolute difference in adjusted mortality or non-home discharge rate, with 95% confidence interval, by operation type.

Compared to *Nonfrail*, the *Frail* cohort had greater observed rates of index-hospitalization mortality, non-home discharge, 30-day unplanned readmission and greater costs for each operative category (S2 Table). Adjusted estimates for each outcome by frail status and operation type were determined following multivariable logistic or linear regression (Table 2). In all operative categories, the *Frail* cohort had significantly greater adjusted rates of mortality at the index hospitalization compared to *Nonfrail*. The greatest absolute difference in adjusted rate of mortality for *Frail* vs *Nonfrail* occurred following repair of perforated ulcer (+8.3%, 95% CI 7.1–10.4%), followed by large bowel resection (+5.9%, 95% CI 5.3–6.5%, Fig 1). Frailty was associated with increased rates of non-home discharge (Fig 1), with greatest differences following repair of perforated ulcer and large bowel resection. Absolute differences in adjusted costs varied by operation, with the greatest difference between *Frail* and *Nonfrail* occurring following repair of perforated ulcer ($24,600, 95% CI 22,300–26,900) and small bowel resection ($21,600, 95% CI $20,000–23,100).

**Table 2:** Adjusted outcomes for all EGS operations by *Nonfrail* and *Frail* cohorts, stratified by operation.

	PUD Repair		Large Bowel Resection		Small Bowel Resection		Lysis of Adhesions		Appendectomy		Cholecystectomy	
	*Nonfrail*	*Frail*	*Nonfrail*	*Frail*	*Nonfrail*	*Frail*	*Nonfrail*	*Frail*	*Nonfrail*	*Frail*	*Nonfrail*	*Frail*
Mortality^a^	6.9 (6.2–7.6)	15.7 (14.2–17.2)	6.0 (5.8–6.3)	11.9 (11.4–12.5)	4.9 (4.6–5.2)	10.7 (10.0–11.5)	1.4 (1.2–1.6)	7.2 (6.2–8.2)	0.4 (0.3–0.5)	3.8 (2.7–4.9)	0.2 (0.2–0.2)	2.3 (2.0–2.6)
Non-Home Discharge^a^	21.0 (19.8–22.1)	64.6 (62.2–67.0)	25.9 (25.2–26.5)	68.1 (67.1–69.0)	18.1 (17.5–18.7)	57.7 (56.2–59.1)	7.0 (6.5–7.4)	43.8 (41.7–46.0)	3.9 (3.6–4.2)	35.9 (33.0–38.8)	2.9 (2.8–3.0)	33.7 (32.7–34.7)
Adjusted Costs^b^	26.1 (25.4–26.9)	50.7 (48.4–53.0)	26.5 (26.0–26.9)	46.8 (45.8–47.8)	24.8 (24.3–25.3)	46.4 (44.8–48.0)	16.9 (16.6–17.3)	37.2 (35.6–38.8)	14.5 (14.2–14.8)	30.8 (29.2–32.4)	11.9 (11.8–12.1)	21.7 (20.8–22.5)
Postoperative LOS^c^	8.0 (7.9–8.3)	14.6 (14.0–15.1)	8.0 (7.9–8.1)	14.0 (13.8–14.3)	7.5 (7.4–7.6)	14.0 (13.6–14.3)	4.9 (4.8–5.0)	11.8 (11.3–12.2)	4.4 (4.3–4.5)	10.6 (10.0–11.2)	2.1 (2.1–2.1)	5.8 (5.6–6.0)
30-Day Readmission^a^	11.4 (10.5–12.3)	19.8 (17.9–21.7)	12.6 (12.2–12.9)	17.6 (16.9–18.3)	12.8 (12.2–13.4)	17.3 (16.2–18.4)	9.5 (9.0–9.9)	16.3 (14.8–17.8)	7.1 (6.7–7.5)	13.7 (11.5–15.9)	5.1 (5.0–5.2)	12.0 (11.3–12.6)

Estimates are derived from logistic or linear regression models with identical covariates as listed in Table 3.

^a^Percentage with 95% CI reported for mortality, non-home discharge, and 30-day readmission rates.

^b^Costs reported in $1000 USD with 95% CI.

^c^Length of stay (LOS) reported as days with 95% CI.

### Factors associated with mortality and readmission following EGS

Multivariable regression identified several additional factors independently associated with mortality and unplanned readmission (Table 3). An Elixhauser Comorbidity Index >2 (adjusted odds ratio, AOR, 1.79, 95% CI 1.65–1.94) and older age (AOR 1.04, 95% CI 1.04–1.04 per 1-year increment) were associated with increased odds of mortality. Specific comorbidities including congestive heart failure, chronic lung disease, ESRD, and chronic liver disease were independently associated with mortality. Nonmetastatic cancer was associated with reduced odds of mortality while there was no association between metastatic cancer and mortality. Notably, transferred patients were at greater odds of mortality (AOR 1.50, AOR 1.28–1.75) while no association was found between mortality and hospital teaching status. Relative to private insurers, Medicare or Medicaid payer status was associated with increased odds of mortality.

**Table 3:** Multivariable models for mortality at index admission and 30-day unplanned readmission.

	In-Hospital Mortality		30-Day Unplanned Readmission	
	Odds Ratio (95% CI)	P-Value	Odds Ratio (95% CI)	P-Value
Age (per 1-year increment)	1.04 (1.04–1.04)	<0.001	0.998 (0.997–0.999)	<0.001
Sex				
Female	0.93 (0.89–0.98)	0.01	0.97 (0.95–0.99)	0.046
Male	Reference		Reference	
Primary Payer				
Medicare	1.45 (1.32–1.59)	<0.001	1.32 (1.27–1.38)	<0.001
Medicaid	1.57 (1.39–1.76)	<0.001	1.31 (1.26–1.37)	<0.001
Other Payer^a^	1.65 (1.44–1.89)	<0.001	1.07 (1.01–1.14)	0.024
Private Insurer	Reference		Reference	
Transfer Status				
Transferred to Operating Hospital	1.50 (1.28–1.75)	<0.001	1.16 (1.03–1.31)	0.013
Non-Transfer	Reference		Reference	
Elixhauser Comorbidity Index				
>2	1.79 (1.65–1.94)	<0.001	1.49 (1.43–1.55)	<0.001
≤2	Reference		Reference	
Comorbidities				
Chronic Liver Disease	3.48 (3.23–3.76)	<0.001	1.14 (1.08–1.20)	<0.001
Chronic Lung Disease	1.32 (1.24–1.40)	<0.001	1.17 (1.13–1.21)	<0.001
Coagulopathy	3.71 (3.47–3.97)	<0.001	1.29 (1.23–1.36)	<0.001
Congestive Heart Failure	1.68 (1.57–1.79)	<0.001	1.25 (1.19–1.31)	<0.001
Coronary Artery Disease	1.06 (1.00–1.13)	0.06	1.17 (1.12–1.22)	<0.001
Diabetes	1.00 (0.94–1.06)	0.928	1.14 (1.11–1.18)	<0.001
End Stage Renal Disease	1.93 (1.74–2.14)	<0.001	1.85 (1.72–1.99)	<0.001
Malignancy				
Metastatic	1.09 (0.99–1.20)	0.089	1.33 (1.25–1.42)	<0.001
Nonmetastatic	0.70 (0.64–0.77)	<0.001	1.09 (1.03–1.16)	0.003
None	Reference		Reference	
EGS Volume				
High	1.00 (0.92–1.08)	0.963	1.08 (1.03–1.13)	0.001
Medium	1.05 (0.98–1.13)	0.158	1.03 (0.99–1.06)	0.165
Low	Reference		Reference	

In addition to listed factors, models include adjustment for teaching status with an interaction term between teaching status and operation type, as well as for frailty, operation type, and the interaction of these terms, with results reported in Table 2.

^a^Other payer includes self-pay, and uninsured.

**Fig 2:**
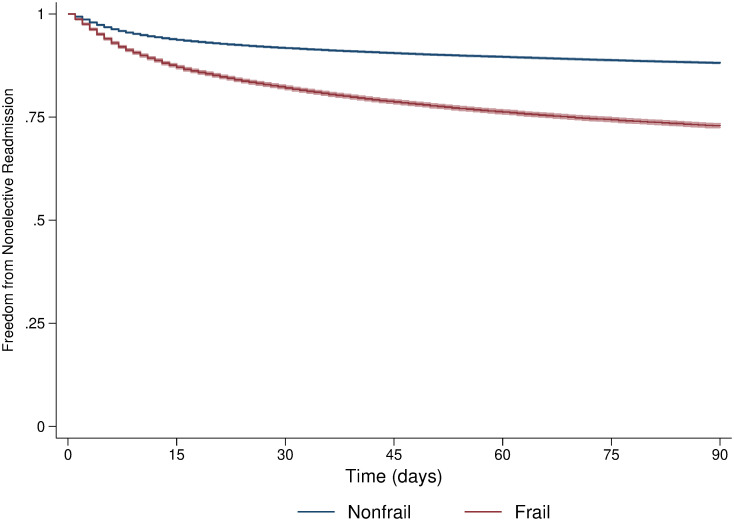
Freedom from unplanned readmission in *Frail* versus *Nonfrail* cohorts. Kaplan Meier curves with 95% confidence interval (shaded) include all EGS operations. Log rank P<0.001.

Among patients who survived to discharge, conditions associated with 30-day unplanned readmission included congestive heart failure, chronic lung disease, and ESRD. Similar to index mortality, the odds of rehospitalization were higher for patients with Medicare and Medicaid coverage when considering private insurance status as reference. Overall, Kaplan-Meier survival analysis showed greater unplanned readmission in *Frail* compared to the *Nonfrail* cohort (Fig 2, log rank P<0.001). When stratified by EGS procedure, frailty remained associated with increased readmission following all EGS operations considered (log rank P<0.001 for each).

## Discussion

**Fig 3:**
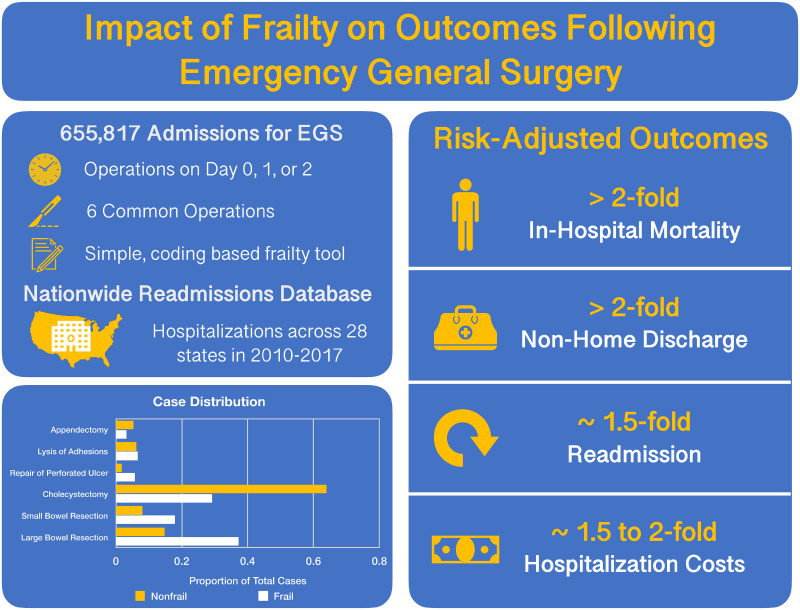
Visual summary of study findings. Frailty adversely impacts odds of in-hospital mortality, non-home discharge, readmission, and is associated with greater resource use.

In this population-based cohort study, we evaluated the impact of frailty, as defined by an administrative coding-based tool, on several clinical and financial endpoints following 6 common EGS operations. Frailty was associated with significantly increased mortality and readmission rates in all operative categories. Moreover, the presence of frailty was associated with a near doubling of adjusted hospitalization costs, with half of the frail cohort was discharged to a nursing home or rehabilitation facility (Fig 3). Importantly, we demonstrated the differential impact of frailty on operative outcomes, with a greater influence noted in more complex operations such as large bowel resection and repair of perforated ulcers.

A large body of literature examining factors influencing outcomes in the EGS population has identified variables such as advanced age, burden of comorbidities as well as physiologic derangements to portend inferior outcomes [2,3,5]. Traditional risk factors aside, many surgeons have considered a patient’s fitness for surgery, informally known as the “eyeball test”, to play an important role in postoperative outcomes. Frailty has been characterized using instruments that range from intricate individualized examinations to scoring based on accumulation of comorbidities [23,24,34]. Given the additional constraints of preoperative testing in EGS, administrative coding algorithms including the NSQIP modified Frailty Index (mFI) have been utilized to assess the presence of frailty in surgical patients. Murphy and colleagues found an association between frailty, defined by the mFI, and 30-day mortality following several common EGS operations [18]. This study, however, was limited to patients aged >40 years with only 3.6% of the cohort considered highly frail. Furthermore, variable associations between the intermediately frail group and index hospitalization outcomes limit the generalizability of the mFI. In contrast to other frailty instruments, derivatives of the ACG indicator used in the present study do not consider traditional risk factors such as heart failure, stroke, and cardiovascular diseases in defining frailty [25,27]. McIsaac et al used the ACG in patients aged >65 years in Ontario, Canada, and found frailty to impact 1-year mortality following cholecystectomy and appendectomy [35]. Our results build on prior work by examining frailty using this binary indicator. The group of administrative codes used in the present study identified 12% of patients as frail and found a consistent adverse impact on outcomes across six common operations in an inclusive, nationally representative, population-based cohort. Importantly, this assessment of frailty captured conditions beyond traditional surgical risk factors, such that frailty remained independently associated with inferior outcomes after adjusting for medical comorbidities. The present study provides new insights into the outcomes expected in this vulnerable population, and may help guide surgical care.

Acuity and severity of illness in EGS present unique challenges in risk factor optimization and perioperative care [2,5]. While frailty may not be a modifiable risk factor in the short term, knowledge of its presence may aid in shared decision-making and counseling regarding expectations. For example, as anastomotic leak greatly increases surgical morbidity, high risk colonic anastomoses may more often warrant proximal diversion in frail patients, as inpatient mortality rates were high for the frail group in the present study. While frailty is often co-existent with medical comorbidities such as coronary disease and chronic lung disease, it remained strongly associated with poor outcomes across all conditions studied, and may provide additional data regarding clinical risk. In particular, frailty may inform discussions regarding expected length of stay, the possibility of discharge to rehabilitation or nursing facilities, and unanticipated rehospitalization. Given the significant cost differential for frail patients, interventions to mitigate complications and facilitate more efficient care may improve outcomes for this group. Similar approaches have been reported by the American College of Surgeons Program for Geriatric Surgery Verification, and have shown promise for improved management of geriatric patients undergoing general surgery operations [36]. Practical measures to reduce common hospital complications that are likely more prevalent in frail patients, such as postoperative delirium, falls, and aspiration, may contribute to improved outcomes. Moreover, aimed at addressing frailty-specific issues that contribute to poor outcomes, such as lack of mobility and poor nutrition, may facilitate the development of evidence-based interventions for frail patients.

Several other variables were also associated with mortality and 30-day readmissions. In the present work, Medicare and Medicaid insurance were associated with greater odds of mortality and readmissions in all EGS categories. Others have reported poor clinical outcomes and increased resource use following elective operations including colectomy and gastrectomy, as well as in the setting of blunt trauma, in these demographic groups [37,38]. These findings may be related to reduced access to care or delays in treatment, and further study of relationships between other social determinants of health, such as education, employment, and race, and access to general surgery care are imperative. Of note, public insurance had a greater magnitude of association with in-hospital mortality and 30-day readmissions compared to several comorbidities including chronic lung disease and heart failure. This underscore the importance of public health efforts to improve access to care, develop strategies for patient engagement, and incentivize hospitals and providers who care for these patients. We similarly found patients who were transferred to the operating hospital to be at increased odds of mortality, which may be related to possible delays of care related to recognition of a surgical condition, referral, and transport time [39]. These factors may be of greater relevance in frail patients, as they are already at greater odds of mortality in all EGS operations.

A unique finding of the present study is the differential impact of frailty on outcomes following various EGS operations. More invasive and complex procedures, such as large bowel resection and repair of perforated ulcer, had a greater absolute difference in adjusted rates of mortality and non-home discharge between the frail and nonfrail cohorts. Conversely, more routine EGS operations, such as cholecystectomy, had the least absolute difference between the two groups. These findings may be related to a greater degree of underlying illness and acute decompensation that result in the need for an urgent colectomy or repair of perforated ulcer. Likewise, reduced tolerance to the physiologic effects of an operative illness and its sequalae may explain the greater differences in non-home discharge rates in complex operations, resulting in the need for further care outside of the hospital. McIsaac et al reported a greater hazard ratio for 1-year mortality with the presence of frailty in patients undergoing appendectomy, cholecystectomy and bowel resection, but not ulcer repair [35]. However, this study did not examine absolute differences in frailty-based outcomes and was limited to patients >65 years of age in a single Canadian province, where practice patterns may vary from the US. While absolute differences were greater for complex operations in this study, even more routine operations such as cholecystectomy had almost a 10-fold increase in adjusted mortality for *Frail* versus *Nonfrail* cohorts. Given the substantial variation in operative risk of these EGS procedures, the present work provides practical guidance that may better inform shared decision making, expectations, and postoperative care.

The present study has several limitations inherent to the nature of a large, administrative database. Although the NRD is the largest, all-payer readmission database, it only estimates approximately 58% of admissions in the United States across 28 states. We limited our analysis to six common operations, and focused on those undergoing surgery on hospital day 0 to 2, which does not encompass the entirety of emergency general surgery. Clinical data, including laboratory values and imaging findings, and measures of disease severity, were unavailable for analysis. Although some aspects of functional status are captured using a coding-based frailty indicator, other clinical markers of frailty, such as hypoalbumenia, sarcopenia, and grip strength, could not be studied. In the NRD, mortality outside of a hospital setting cannot be identified, and, as such, we limited our analysis to readmissions. Despite these limitations, we used validated data practices recommended by HCUP to report nationally representative outcomes of EGS.

## Conclusions

We found frailty to be independently associated with inferior outcomes following all examined EGS operations, with increased rates of mortality, non-home discharge, and hospitalization costs. Frailty has the most pronounced absolute effect on outcomes for more complex and higher acuity operations such as repair of perforated ulcer or large bowel resection, and the least for routine operations. These findings underscore the value of a simple frailty assessment for patients undergoing EGS operations, which may be useful in setting expectations about courses of care as well as in identifying patients that may require more intensive care postoperatively. Further study to identify factors that may mitigate the effect of frailty, as well as efforts to implement practices to better manage frail patients perioperatively, may improve outcomes for this vulnerable patient population.

## Supporting information

S1 Table Derivatives of Johns Hopkins ACG categories and representative diagnoses. (DOCX)

S2 Table Unadjusted outcomes for *Nonfrail* and *Frail* cohorts stratified by operation. ^a^Costs reported in $1000 USD with IQR. ^b^Length of stay reported as days with IQR. P<0.001 for all comparisons between *Frail* versus *Nonfrail* by operation and outcome.
(DOCX)
