# Supplementary material for: Impact of frailty on clinical outcomes and resource use following emergency general surgery in the United States
Source: PLoS One. 2021 Jul 23;16(7):e0255122. doi: 10.1371/journal.pone.0255122 (PMC8301636; doi:10.1371/journal.pone.0255122)
Supplement: S1 Table — (DOCX) [file pone.0255122.s001.docx]

**S1 Table.** **Derivatives of Johns Hopkins ACG Categories and representative diagnoses.**

| **Category** | **Representative Diagnoses** | **ICD-10-CM Codes** |
| --- | --- | --- |
| Malnutrition | Nutritional marasmus; severe protein-calorie malnutrition | E41, E43, E44, E45, E46 |
| Dementia | Alzheimer's disease; frontotemporal dementia; unspecified dementia | F01, F02, F03, F05, G30, G31.0 |
| Severe Vision Impairment | Legal blindness; Blindness, both eyes | H54.0X, H54.1, H54.8 |
| Decubitus Ulcer | Decubitus ulcer | L89 |
| Urinary incontinence | Incontinence without sensory awareness; continuous leakage; mixed incontinence | N31, N36.4, N39.42, N39.45, N39.46 |
| Fecal incontinence | Fecal incontinence | R15 |
| Weight loss | Abnormal weight loss; adult failure to thrive | R62.7, R63.0, R63.3, R63.4 |
| Social support needs | Inadequate housing; confined mobility | Z59.0, Z59.1, Z59.4, Z59.7, Z59.8, Z59.9, Z74, Z75.0, Z75.1, Z75.3, Z75.4, Y93E, Y93.F, Y93.G |
| Difficulty in Walking | Abnormalities of gait and mobility; difficulty in walking | R26, R27, Z99.3 |
| Falls | Falls on and from stairs and steps; fall on same level | W00.0XXA, W00.1XXA, W00.2XXA, W00.9XXA, W01.0XXA, W01.10XA, W01.110A, W01.111A, W01.118A, W03.XXXA, W04.XXXA, W05.0XXA, W05.2XXA, W06.XXXA, W07.XXXA, W08.XXXA, W10.0XXA, W10.1XXA, W10.2XXA, W10.8XXA, W10.9XXA, W17.81XA, W17.89XA, W18.00XA, W18.01XA, W18.02XA, W18.09XA, W18.11XA, W18.12XA, W18.31XA, W18.39XA, W18.40XA, W18.41XA, W18.42XA, W18.49XA, W19.XXXA |
