# Supplementary material for: Impact of frailty on clinical outcomes and resource use following emergency general surgery in the United States
Source: PLoS One. 2021 Jul 23;16(7):e0255122. doi: 10.1371/journal.pone.0255122 (PMC8301636; doi:10.1371/journal.pone.0255122)
Supplement: S2 Table — aCosts reported in $1000 USD with IQR. bLength of stay reported as days with IQR. P<0.001 for all comparisons between Frail versus Nonfrail by operation and outcome. (DOCX) [file pone.0255122.s002.docx]

|  | **PUD Repair** | | **Large Bowel Resection** | | **Small Bowel Resection** | | **Lysis of Adhesions** | | **Appendectomy** | | **Cholecystectomy** | | |
| --- | --- | --- | --- | --- | --- | --- | --- | --- | --- | --- | --- | --- | --- |
|  | **Nonfrail** | **Frail** | **Nonfrail** | **Frail** | **Nonfrail** | **Frail** | **Nonfrail** | **Frail** | **Nonfrail** | **Frail** | **Nonfrail** | **Frail** |  |
| Mortality | 715  (7.1) | 697  (15.5) | 5,203  (6.1) | 3,488  (12.0) | 2,290  (4.9) | 1,499  (10.7) | 528  (1.5) | 378  (7.3) | 134  (0.4) | 99  (4.0) | 787  (0.2) | 548  (2.4) |  |
| Non-home Discharge | 1,656  (21.2) | 1,838  (64.2) | 14,259  (25.9) | 12,162  (67.7) | 6,465  (18.2) | 5,202  (57.5) | 2,175  (7.0) | 1,526  (44.0) | 1,079  (3.9) | 638  (35.7) | 10,240  (2.9) | 6,145  (33.8) |  |
| Costs^a^ | 18.8  (12.9-30.0) | 34.5  (21.6-60.4) | 20.6  (15.0-30.1) | 33.4  (21.7-54.7) | 18.8  (13.3-28.2) | 31.6  (20.2-52.4) | 13.1  (9.3-19.6) | 25.0  (16.1-43.4) | 11.5  (8.2-16.9) | 22.3  (13.6-37.7) | 10.0  (7.6-13.7) | 15.0  (10.5-23.7) |  |
| LOS^b^ | 7  (5-10) | 12  (8-19) | 7  (5-10) | 12  (8-19) | 7  (5-10) | 11  (7-18) | 4  (3-7) | 10  (6-16) | 4  (2-6) | 9  (5-14) | 3  (2-4) | 5  (3-8) |  |
| Postoperative LOS^b^ | 6  (4-9) | 11  (7-19) | 7  (5-9) | 11  (7-18) | 6  (4-9) | 11  (7-17) | 4  (2-6) | 9  (5-15) | 3  (2-6) | 8  (5-14) | 1  (1-2) | 4  (2-7) |  |
| 30-Day Readmission | 1,085  (11.5) | 752  (19.8) | 10,054  (12.6) | 4,461  (17.4) | 5,688  (12.8) | 2,138  (17.1) | 3,341  (9.5) | 863  (17.9) | 2,174  (7.1) | 325  (13.7) | 18,943  (5.1) | 2,691 (12.1) |  |

**S2 Table. Unadjusted outcomes for *Nonfrail* and *Frail* cohorts stratified by operation.**

^a^Costs reported in $1000 USD with IQR. ^b^Length of stay reported as days with IQR. P<0.001 for all comparisons between *Frail* versus *Nonfrail* by operation and outcome.
